# Endocytosis, Distribution, and Exocytosis of Polystyrene Nanoparticles in Human Lung Cells

**DOI:** 10.3390/nano13010084

**Published:** 2022-12-24

**Authors:** Yuan-Yuan Liu, Jie Liu, Hao Wu, Qiangqiang Zhang, Xue-Rui Tang, Dan Li, Chen-Si Li, Yuanfang Liu, Aoneng Cao, Haifang Wang

**Affiliations:** 1 Institute of Nanochemistry and Nanobiology, Shanghai University, Shanghai 200444, China; 2 Beijing National Laboratory for Molecular Sciences, College of Chemistry and Molecular Engineering, Peking University, Beijing 100871, China

**Keywords:** nanoplastic, polystyrene particles, lung cells, cellular uptake, translocation, excretion

## Abstract

Nanoplastics, one component of plastic pollution, can enter human bodies via inhalation and thus threaten human health. However, the knowledge about the uptake and exocytosis of nanoplastics in cells of human lung organs is still very limited. Herein, we investigated the endocytosis, distribution, and exocytosis of polystyrene nanoparticles (PS NPs) of 50 nm (G50PS) and 100 nm (R100PS) in A549 cells and BEAS-2B cells. We found that both the cellular uptake of PS NPs increased positively with exposure time and dose, and A549 cells ingested more PS NPs than BEAS-2B cells did. In addition, the intracellular content of G50PS was higher than that of R100PS except at a higher dose and longer time. The ingested PS NPs were distributed mainly in lysosomes, while many G50PS appeared around the cell membrane, and R100PS also accumulated in mitochondria in BEAS-2B cells. As for the exocytosis, R100PS was more difficult to excrete than G50PS. Lysosomes in A549 cells and actin and microtubule in BEAS-2B cells were involved in the exocytosis of the PS NPs. These findings provide detailed information about the translocation of nanoplastics in lung cells, which is valuable for the safety assessment of nanoplastics in the environment.

## 1. Introduction

A huge amount of plastic is produced every year and used in various fields. Subsequently, a large amount of plastic waste is generated and discarded into the environment, and part of the plastics are degraded or decomposed into microplastics and then into nanoplastics through natural weathering [1]. Microplastics and nanoplastics have spread all over the world and are found in water, air, our food, and even the human body [2,3,4,5]. Due to their small size and easier transportation, the potential health effect of nanoplastics has recently attracted extensive attention [3]. However, the detailed mechanism of the interaction of nanoplastics and cells is not fully understood.

In plastics, polystyrene (PS) is widely used in the production of compact discs, toys, toothbrushes, etc., because of its high transparency, good wear resistance, and easy dyeability [6]. In addition, due to its low cost and convenient use, PS also appears in food packaging bags, clothing, kitchenware, auto parts, construction, etc. [7]. Most PS products undergo an inevitable wear process and thus produce a large amount of PS nanoparticles (PS NPs), some of which PS NPs may enter the air. For example, textiles have different degrees of abrasion during washing [8,9,10,11], drying [10,11], and wearing [12], resulting in PS NPs in the air [4,13,14]. Therefore, in addition to ingestion and skin contact, PS NPs also can enter organisms via inhalation [6,15,16], notably occupational inhalation during the production and processing of PS NPs. 

When PS particles enter the human respiratory system, they first encounter airway mucus, which eliminates large PS particles [17]. Among those not eliminated, pulmonary macrophages may phagocytize 1-3 μm PS particles, and the small PS particles get into the bronchi and alveoli [18]. It was reported that NPs could travel from the lung to the bloodstream and lymph nodes [19], but NPs of more than 50 nm could reside within the lung for a longer time [20]. Therefore, it is important to study the interaction of PS NPs with sizes of around 50 nm and typical cells of bronchi and alveoli. 

There are already many studies on the interaction between PS NPs and human cells, but most of these studies focus on the cytotoxicity of PS NPs [21,22,23,24,25,26,27]. Studies show that PS NPs could produce reactive oxygen species [21,24,27], cause a series of inflammatory reactions [26,28], and reduce cell viability [25,28]. PS NPs could also induce apoptosis and inflammation of A549 cells and induce epithelial-to-mesenchymal transition in A549 cells, inferring that PS NPs could increase the risk of pulmonary fibrosis and cancer [29]. Studies also show that nano-sized PS was more cytotoxic and genotoxic to A549 cells than micro-sized PS [29], and 25 nm PS NPs were easier to be ingested by A549 cells and showed higher toxicity potential than 70 nm PS NPs [30]. Generally, the content and location of NPs in cells are critical for the biological effects of NPs [31,32,33,34]. Moreover, the content of NPs in cells not only depends on the cellular uptake but also is related to the exocytosis of NPs [35], in which the size of NPs is a critical influencing factor [27,30]. Thus, detailed information about the cellular uptake, distribution, and exocytosis of PS NPs is crucial for understanding their biological effects and facilitating the safety assessment of nanoplastics, which is still missing.

In this study, we selected PS NPs of 50 nm and 100 nm and typical lung cells of bronchi and alveoli, i.e., human bronchial epithelial cells (BEAS-2B) and human alveolar epithelial cells (A549), as the models to investigate the whole process of cellular uptake, distribution, and exocytosis of nanoplastics, and thus provide an in-depth understanding of the interaction of nanoplastics and lung cells. This information may help producers and users assess the safety of PS nanoplastics accurately and thus guide the design, production, and application of PS products.

## 2. Materials and Methods

### 2.1. PS NPs

Fluorescence-labeled PS NPs with a diameter of around 50 nm (green, denoted as G50PS) and around 100 nm (red, denoted as R100PS) were purchased from Thermo Fisher Scientific, Waltham, MA, USA). The morphology and size of the PS NPs were characterized using TEM (HT7700, Hitachi, Tokyo, Japan). The hydrodynamic size and ζ-potential of the PS NPs in aqueous solutions were determined using a Nano ZS90 (Malvern Instruments, Malvern, UK). The PS NPs were suspended in water or cell culture medium (DMEM) containing 10% (*v/v*) fetal bovine serum (FBS, Sigma-Aldrich, NJ, USA) at a concentration of 40 μg/mL, and the PS NPs suspensions were kept in a humidified incubator (37 °C, 5% CO_2_/95% air) for 0, 6, and 12 h.

The fluorescence spectra of the PS NPs were recorded using a fluorescence spectrometer (F7000, Hitachi, Tokyo, Japan). We should note here, although the optimal excitation wavelengths given in the product specification are 468 nm for G50PS and 542 nm for R100PS, limited by the configuration of the flow cytometer, 488 nm was adopted in all the measurements of cellular fluorescence intensity in this study. The fluorescence stability of the PS NPs was investigated following the literature [36]. Briefly, 5 mL of 0.2 mg/mL PS NPs dispersed in the medium containing 10% FBS was incubated in the incubator for 0, 1, 2, 4, 6, and 12 h. Next, the PS NPs suspensions were transferred to ultrafiltration tubes (Amicon ultra-15 10K, Millipore Co., Billerica, MA, USA) and centrifuged (5000 rpm) for 30 min. The filtrates (200 μL) were collected and their fluorescence intensities were measured using a microplate reader (Varioskan flash, Thermo Fisher Scienticific, Waltham, MA, USA). The fluorescence stability of PS NPs is expressed with the fluorescence leakage of PS NPs.

### 2.2. Cell Culture and Cytotoxicity Evaluation

Human bronchial epithelial cell line BEAS-2B was kindly provided by Dr. Baoxin Bai of Shanghai East Hospital, China. Human lung carcinoma epithelial cell line A549 (ATCC, No: CCL-185) was obtained from the Cell Bank of Type Culture Collection of the Chinese Academy of Sciences (Shanghai, China). Both kinds of cells were cultured in high glucose DMEM (4.5 g/L glucose) supplemented with 10% (*v/v*) FBS and 1% penicillin–streptomycin (hereinafter called culture medium) at 37 °C and 5% CO_2_ in the incubator. Cells were seeded into 96-well plates (8 × 10^3^ cells per well, for the cell viability assay), 12-well plates (5 × 10^4^ cells per well, for the flow cytometry analysis), or dishes (d = 35 mm, 2 × 10^5^ cells per dish, for the confocal laser scanning microscopic (CLSM) investigation), and incubated overnight for the following assays. 

To evaluate the cytotoxicity of PS NPs, the cell counting kit-8 (CCK-8; Dojindo, Kyushu, Japan) was used following the manufacturer’s instructions [35]. Briefly, the cells in 96 plates were exposed to the culture medium containing the PS NPs of different concentrations for 24 h. Subsequently, the culture medium was abandoned, and the CCK-8 solution (100 μL, containing 10% WST-8) was added. The optical density of each well was recorded at 450 nm on a microplate reader (Varioskan Flash, Thermo Fisher Scienticific, Waltham, MA, USA) after 1 h incubation. 

### 2.3. Cellular Uptake of PS NPs

To study the concentration effect of PS NPs on their cellular uptake, cells were cultured in the culture medium containing G50PS or R100PS of different concentrations for 2 h. To study the time effect on the cellular uptake of PS NPs, cells were cultured in the culture medium containing 20 μg/mL of G50PS or R100PS for different times (0, 1, 2, 4, 6, 12, and 24 h). After incubation, the cells were washed with cold D-Hanks buffer three times, detached and counted, and subsequently suspended in 300 μL of 4% paraformaldehyde for the flow cytometry analysis (CytoFLEXs, Beckman Coulter Inc., CA, USA, Ex 488 nm). The cell-averaged uptake of PS NPs is represented by the mean fluorescence intensity (FI) of 10000 cells (after subtraction of the FI of the control), and the total cellular uptake of PS NPs is calculated by multiplying the mean FI by the corresponding cell number counted. 

To compare the cellular uptake capacity between G50PS and R100PS, the external standard method [28,37] was used to calibrate the fluorescence results in cells measured using flow cytometry. Briefly, after incubation with PS NPs (20 μg/mL) for 4 h, the cells were washed and collected by centrifugation and then crushed using the cell lysis buffer (P0013, Beyotime Biotechnology Co., Ltd., Shanghai, China). Standard solutions containing different concentrations of the PS NPs were added separately to the corresponding lysates to prepare the PS solutions containing ingested PS NPs plus 0.5 μg/mL, 1 μg/mL, or 2 μg/mL of PS NPs. Afterward, the fluorescence intensity of these solutions was measured via an FS5 fluorescence spectrometer (Edinburgh Instruments, Edinburgh, UK) to obtain the contents of G50PS and R100PS in cells. Further, these values were used to calibrate the results measured by flow cytometry to obtain the FI ratio of the two PS NPs at equal mass inside a cell.

To understand the mechanisms of the cellular uptake of PS NPs, the cellular uptake at 4 °C and in the presence of various endocytosis inhibitors was measured [35]. Briefly, cells were pre-incubated at 4 °C or 37 °C (the control) for 30 min and then incubated in the culture medium containing PS NPs at the same temperature for 4 h. Subsequently, the cells were collected and measured by flow cytometry as described above. In addition, cells were pre-incubated in the culture medium containing an inhibitor for 30 min. The inhibitors included 7 μg/mL chlorpromazine hydrochloride (CPZ) (Innochem Co., Ltd., Beijing, China) and 100 μM Amantadine (Shanghai Aladdin Biochemical Technology Co., Ltd., Shanghai, China) for clathrin-mediated endocytosis, 2.5 μg/mL Filipin III (APExBIO Technology LLC, Houston, TX, USA) and 2 mM MβCD (Sigma-Aldrich, NJ, USA) for caveolae-mediated endocytosis, and 50 μM Amiloride (Innochem Co., Ltd., Beijing, China) for micropinocytosis [38,39,40]. Next, the culture medium was discarded and the fresh culture medium containing the same inhibitor plus 20 μg/mL of PS NPs was added to the well. After 4 h incubation, the culture medium was abandoned again and the cells were treated and detected by flow cytometry, as described above.

### 2.4. Locations of PS NPs in Cells

Cells were incubated in the culture medium containing 20 μg/mL PS NPs for 2, 4, 12, and 24 h, and then the medium was abandoned. Subsequently, the cells treated with G50PS were exposed to 100 nM of LysoTracker-Red DND-26 (Ex 577 nm/Em 590 nm) or MitoTracker-Red FM (Ex 581 nm/Em 644 nm) in the D-Hanks solution for 30 min, while the cells treated with R100PS were exposed to 100 nM of LysoTracker-Green DND-26 (Ex 504 nm/Em 511 nm)( Thermo Fisher Scienticific, Waltham, MA, USA) or 100 nM of MitoTracker-Green FM (Ex 490 nm/Em 516 nm) (Thermo Fisher Scienticific, Waltham, MA, USA) in the D-Hanks solution for 30 min. Finally, the cells were washed and imaged under a CLSM (FM 1000, Olympus, Tokyo, Japan).

### 2.5. Exocytosis of PS NPs

Cells were incubated firstly with 20 μg/mL PS NPs for 4 h, and then the medium was removed, and the cells were washed with the D-Hanks solution three times. Afterward, the cells were cultured in the fresh culture medium for 0, 0.5, 1, 2, 4, and 6 h. The cell-averaged intracellular content of PS NPs (Residue per cell, in the percentage of the initial content in the cells) was obtained by averaging FI values of 10,000 cells, and the amount of PS NPs released into extracellular culture medium (Exocytosis, in the percentage of the initial total amount in all cells) was calculated by subtracting the total intracellular content at the time of exocytosis (calculated by multiplying the mean FI by the cell number counted at the time of exocytosis) from the initial total intracellular content (calculated by multiplying the mean FI by the cell number counted at 0 h).

The excretion pathways of PS NPs from cells were investigated by incubating the cells for 2 h in the presence of different exocytosis inhibitors at 37 °C following the procedure reported previously [35]. In short, cells were pre-incubated with the culture medium containing 20 μg/mL PS NPs for 4 h. Then, the cells were washed with the D-Hanks solution three times and cultured in fresh culture medium at 4 °C or 37 °C for 2 h, or in the culture medium containing an exocytosis inhibitor for 2 h. The inhibitors were Brefeldin A (89 mM) (Beyotime Biotechnology Co., Ltd., Shanghai, China), Monensin (48 mM) (Beyotime Biotechnology Co., Ltd., Shanghai, China), LY294002 (1 mM) (Beyotime Biotechnology Co., Ltd., Shanghai, China), Vacuolin-1 (5 μM) (Selleck Chemicals LLC, Houston, TX, USA), Bafilomycin A1 (100 nM) (Abcam Plc, Cambridge Science Park, UK), CytoD (5 μM) (Shanghai Aladdin Biochemical Technology Co., Ltd., Shanghai, China), Nocodazole (15 μM) (Shanghai Aladdin Biochemical Technology Co., Ltd., Shanghai, China), and Filipin III (15 μM) (APExBIO Technology LLC, Houston, TX, USA) [39,41,42]. Afterward, the intracellular contents of PS NPs in the cells were detected by flow cytometry, as described above.

The exocytosis mechanism of PS NPs was further investigated through CLSM. Cells were pre-incubated with 20 μg/mL PS NPs for 4 h. Then, the medium was removed, and the cells were washed with the D-hanks solution three times. After that, the cells were cultured in fresh culture medium for 0, 2, and 4 h. The medium was removed, and the cells were incubated in the D-Hanks solution containing 100 nM of LysoTracker DND-26 (Green for R100PS and red for G50PS) or 100 nM of MitoTracker FM (Green for R100PS and red for G50PS) for 30 min. Finally, the cells were imaged on the CLSM.

### 2.6. Statistical Analysis

All means were calculated from three independent experiments, and the data are expressed as the mean ± standard deviation. One-way analysis of variance (ANOVA) was used to test the statistical significance of differences among the control and treated groups. A value of *p* < 0.05 was considered to be statistically significant.

## 3. Results

### 3.1. PS NP Samples

**Figure 1:**
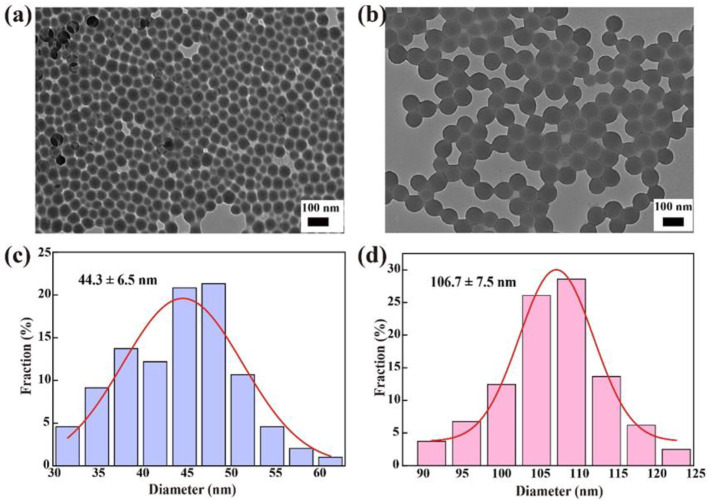
TEM images of G50PS (**a**) and R100PS (**b**) and the corresponding particle size statistics of G50PS (**c**) and R100PS (**d**).

The PS NPs used in this study, G50PS and R100PS, are spherical (Figure 1a,b), and their average sizes are 44.3 nm for G50PS (Figure 1c) and 106.7 nm for R100PS (Figure 1d). The hydrodynamic size and ζ-potential measurements confirmed that the uniform and stable dispersions of PS NPs were formed in ultrapure water and culture medium. As shown in Table 1, the mean hydrated particle sizes of G50PS and R100PS in water were 40.3 nm and 86.9 nm, respectively, and their ζ-potentials were highly negative. In culture medium, the mean hydrodynamic sizes of G50PS and R100PS increased to 62.8 nm and 103.0 nm, respectively, and the ζ-potential of both decreased to about –10 mV. The hydrodynamic sizes and potentials of the PS NPs were not significantly affected by culture time in both water and culture medium.

Figure S1 shows the fluorescence spectra of the PS NPs excited at 488 nm. The emission peaks of G50PS and R100PS were at about 508 nm and 578 nm, respectively. The fluorescence intensity (FI) of G50PS and R100PS increased linearly with their concentrations, respectively, in both water and the culture medium (Figure S2), indicating that the fluorescence in PS particles was uniform.

In addition, the fluorescence labeling of the PS NPs was stable. As shown in Figure S3, the fluorescence leakage of G50PS incubated in culture medium at 37 °C was less than 3% in 6 h and 5% in 12 h. R100PS had no obvious fluorescence leakage, either, as indicated by a leakage ratio of less than 3% in 12 h. 

These results indicated that the two PS NPs were monodispersed in aqueous solutions and had good fluorescence stability under current experimental conditions, which ensures the reliability of subsequent cell experiments.

### 3.2. Cellular Uptake of PS NPs

We evaluated the cell viability of the PS NPs using the CCK-8 kits before cellular uptake experiments. As shown in Figure S4, after 24 h exposure, BEAS-2B cells were more susceptible to the PS NPs than A549 cells, in accordance with previous results of multifarious nanomaterials on the two kinds of cells [43,44,45]. The viabilities of A549 and BEAS-2B cells were still higher than 87% after exposure to 160 μg/mL PS NPs, indicating that G50PS and R100PS were non-toxic under the experimental conditions. To ensure the cellular uptake and exocytosis properties of the cells were not affected, 20 μg/mL PS NPs (cell viability was higher than 90% after 24 h) were used in the main parts of cell experiments. 

**Figure 2:**
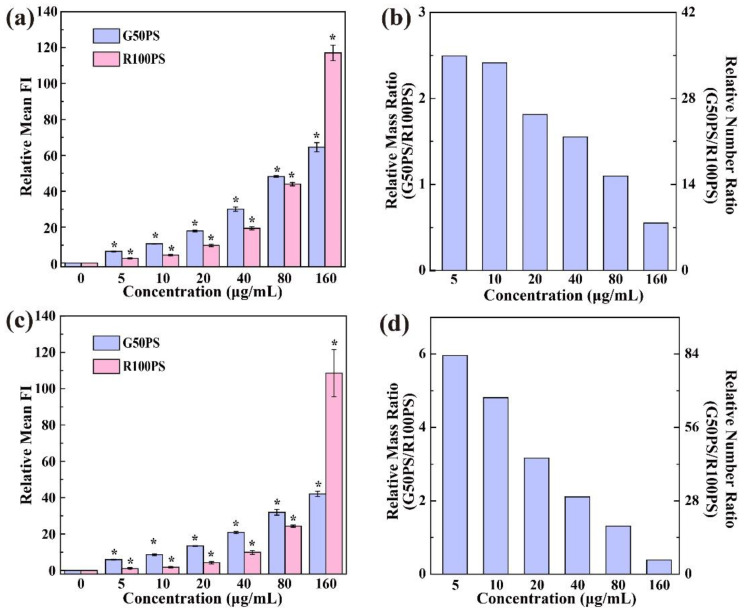
Cellular uptake of PS NPs after cells were incubated with PS NPs of different concentrations for 2 h. The uptake is represented as the relative content of PS NPs in cells (**a**,**c**) and the relative mass (left y-axis)/number (right y-axis) ratio (G50PS/R100PS) of PS NPs in cells (**b**,**d**). (**a**,**b**) A549 cells. (**c**,**d**) BEAS-2B cells. The content of R100PS in BEAS-2B cells incubated with 5 μg/mL R100PS was set as 1 (**a**,**c**). * *p* < 0.05 vs. the control (n = 3).

The cellular uptake of PS NPs was quantified by flow cytometry. Figure S5 shows that the mean fluorescence intensity (FI) in A549 and BEAS-2B cells increased with the increase in the concentration of PS NPs, suggesting that the intracellular contents of PS NPs correlated positively with their concentrations, which is consistent with previous studies [22,35,46]. To compare the cellular uptake capacity between G50PS and R100PS, the contents of the two PS NPs in BEAS-2B cells after exposure to 20 μg/mL PS NPs for 4 h were measured by the external standard method (Figure S6a). The results indicate that 0.42 μg/mL of G50PS and 0.14 μg/mL of R100PS in culture medium were ingested by BEAS-2B cells (Figure S6b). In other words, about 2.1% of G50PS exposed and 0.7% of R100PS exposed were internalized by BEAS-2B cells within 4 h. Using these values to calibrate the FIs measured by flow cytometry, the FI ratio of the two PS NPs at equal mass inside a cell was calculated as G50PS:R100PS = 11.85 (Figure S6c). Using this ratio and assuming that the PS NPs are standard spheres (G50PS: 44.3 nm; R100PS: 106.7 nm), the relative mass/number of intracellular G50PS/R100PS could be calculated accordingly (Figure 2) from the FIs measured by flow cytometry (Figure S5). 

As shown in Figure 2a,c, the intracellular contents of R100PS increased more rapidly than that of G50PS. In both kinds of cells, the mass contents of G50PS in cells were always higher than that of R100PS except at the concentration of 160 μg/mL, where the mass content of R100PS surpassed that of G50PS. Many studies reported that cells displayed a higher ability to ingest particles of 50 nm than those of 100 nm [37,47]. However, our results indicate that this phenomenon is only valid within a specific concentration range. Figure 2b,d display the relative mass and number ratios of intracellular G50PS/R100PS in A549 and BEAS-2B cells, respectively. The results clearly showed that the higher the concentration of PS NPs, the lower the intracellular ratios of G50PS/R100PS. In A549 cells, the intracellular mass/number of G50PS to R100PS decreased from 2.49/34.86 to 0.55/7.70 when the PS NPs concentration increased from 5 μg/mL to 160 μg/mL. In other words, the uptake number of G50PS was much higher than that of G100PS at all tested concentrations. Moreover, generally, more PS NPs were ingested by A549 cells than by BEAS-2B cells, regardless of the size of PS. This finding is in accordance with previous studies on other particles [43,45], yet, different results are also reported [48]. In addition, it was found that the uptake ratio of G50PS to R100PS at lower concentrations in BEAS-2B cells was much higher than that in A549 cells, but the gap narrowed with the increase in concentration, and at 160 μg/mL PS NPs, the uptake mass ratio in BEAS-2B cells exceeded that in A549 cells. The A549 cell line is originated from the human alveolar epithelium and maintains many biochemical characteristics of pneumocytes type II [49], while the BEAS-2B cell line is originated from the human bronchial epithelium and is important in the maintenance of mucosal integrity [50]. They come from different tissues and possess different biological properties. In addition, BEAS-2B is a less manipulated non-tumor cell line, making it more vulnerable to the damage of reactive oxygen, while A549 is a transformed cancer cell line and can secrete pulmonary surfactants [49], which may change the biochemical environment around cells and the properties of NPs by adsorption. Therefore, the difference between A549 cells and BEAS-2B cells may be the mixed effects of these factors. In addition, the properties of different NPs could also be an influencing factor. For example, Reczynska et al. found that BEAS-2B cells internalized a significantly higher amount of SiO_2_-coated SPION (Superparamagnetic iron oxide NPs) than A549cells, while no significant difference was observed between the two kinds of cells treated with unmodified SPIONs [48]. 

**Figure 3:**
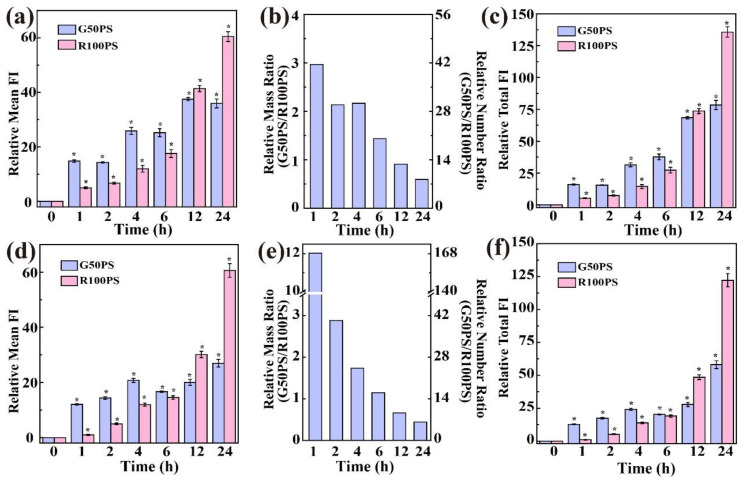
Cellular uptake of PS NPs after cells were exposed to PS NPs (20 μg/mL) for different times. The uptake is represented as the relative content of PS NPs in cells (**a**,**d**), the relative mass (left y-axis)/number (right y-axis) ratio (G50PS/R100PS) of PS NPs in cells (**b**,**e**), and the relative total content of intracellular PS NPs calculated by multiplying relative mean FI with cell number (**c**,**f**). (**a**–**c**) A549 cells. (**d**–**f**) BEAS-2B cells. The content of R100PS in BEAS-2B cells incubated with 20 μg/mL R100PS for 1 h was set as 1 (**a**,**d**). * *p* < 0.05 compared with the control (n = 3).

The uptake of the PS NPs by the two kinds of cells was not only dose-dependent but also time-dependent. As shown in Figure 3a,d, and Figure S7a,d, the content of R100PS in both kinds of cells and that of G50PS in BEAS-2B cells displayed an increasing trend without reaching saturation within 24 h, while that of G50PS in A549 reached saturation at 12 h (Figure 3a and Figure S7a). In addition, we found a slight decrease in the content of G50PS at 6 h in both kinds of cells, which may be caused by cell division. Unlike our results, the cellular uptake of 90 nm PS in the zebrafish cell line ZF4 reached a peak within 2 h and then decreased [51]. The difference possibly comes from different PS NPs and cell lines. 

As for the uptake ratios of G50PS/R100PS in the two kinds of cells, both the mass and number ratios decreased over time; especially, there was a huge decrease in BEAS-2B cells from 1 h to 2 h (Figure 3e). The decreased ratios suggest that the content increase in R100PS in cells was more rapid than that of G50PS as time went on. For example, in A549 cells, the mass/number content of G50PS was 2.97/41.58 times that of R100PS at 1 h; nevertheless, it dropped to just 0.59/8.26 times that of R100PS at 24 h (Figure 3b). That is, the two kinds of cells ingested more G50PS within a short time period but ingested more R100PS at longer exposure time (Figure 3a,b,d,e), while the turning point was at 12 h. Thus, the conclusion that cells ingest more NPs at about 50 nm than NPs at about 100 nm is only valid under a certain concentration range and exposure time. Comparing the uptake capability between the two kinds of cells, A549 cells were still more likely to ingest PS NPs, especially G50PS, than BEAS-2B cells, possibly because the NPs of 50 nm have the fastest wrapping time and thus are easy to be endocytosed [47], and pulmonary surfactants secreted by A549 cells [49] could be adsorbed onto PS NPs and thus changed the properties of the PS NPs presented.

Considering that cell proliferation causes a decrease in the intracellular content of NPs, we counted the cell numbers at different time points to calibrate the total uptake of PS NPs in cells. Because the cell number increased with time and there were no evident differences between the cells treated with the two PS NPs (Figure S7b,e), the total uptake of PS NPs by the two kinds of cells, including G50PS in A549 cells (reached saturation based on cell-averaged content), increased accordingly, and did not reach the maximum at 24 h (Figure 3c,f). At the same time, the total mass and number ratios of G50PS/R100PS remained virtually unchanged compared with the corresponding ratios based on the cell-averaged results (Figure 3b vs. Figure S7c, Figure 3e vs. Figure S7f).

### 3.3. Endocytosis Mechanism of PS NPs

To reveal the detailed mechanisms of the uptake of PS NPs by cells, we selected three classes of endocytosis inhibitors, namely two clathrin-mediated endocytosis inhibitors (CPZ and Amantadine), two caveolae-mediated endocytosis inhibitors (Filipin III and MβCD), and one macropinocytosis inhibitor (Amiloride) to selectively block endocytosis pathways of the two PS NPs during the cellular uptake experiments. The inhibitors at the test conditions were confirmed to be non-toxic (Figure S8).

**Figure 4:**
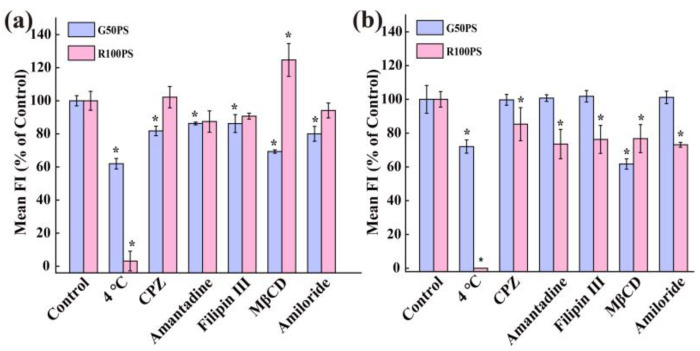
Mechanisms involved in the uptake process of PS NPs by A549 cells (**a**) and BEAS-2B cells (**b**) * *p* < 0.05 compared with the control, (n = 3).

Firstly, we investigated whether the internalization of the PS NPs was an energy-dependent process or not. As shown in Figure 4, the uptake of G50PS by the cells was partly inhibited at 4 °C, and that of R100PS was almost completely inhibited at 4 °C, indicating that the uptake of R100PS required more energy than that of G50PS [52]. The energy-independent internalization of 50 nm NPs has been reported [53,54], and such internalization is possibly due to the partition of PS NPs in the water-phospholipid system [53]. 

Next, the effects of inhibitors on the cellular uptake of PS NPs were investigated. Figure 4a shows that all three classes of inhibitors reduced the uptake of G50PS by A549 cells; that is, the clathrin-mediated endocytosis, caveolae-mediated endocytosis, and macropinocytosis all contributed to the uptake of G50PS by A549 cells. In addition, MβCD showed the greatest ability to inhibit the uptake of G50PS (69% of the control), suggesting that caveolae-mediated endocytosis played an important role in the uptake of G50PS by A549 cells. Zhang et al. observed 70 nm PS entered A549 cells and Caco-2 cells via all three kinds of uptake pathways [16], and Ding et al. found that the caveolae-mediated endocytosis was the main uptake pathway for 50 nm PS in GES-1 cells [55]. In addition, clathrin-mediated endocytosis and caveolae-mediated endocytosis were also reported to be the main uptake pathways for 50 nm NPs in different cells [53,54]. However, R100PS behaved much differently. The inhibitors, especially MβCD, did not significantly inhibit the uptake of R100PS by A549 cells (Figure 4a), inferring that there were other energy-dependent endocytosis pathways, such as the clathrin- and caveolae-independent endocytosis, which is much different from the result of Yang et al. [51]. In BEAS-2B cells, the caveolae-mediated uptake was the main pathway for G50PS, while the clathrin-mediated endocytosis, caveolae-mediated endocytosis, and macropinocytosis contributed comparably to the uptake of R100PS (Figure 4b). 

### 3.4. Subcellular Localization of PS NPs

**Figure 5:**
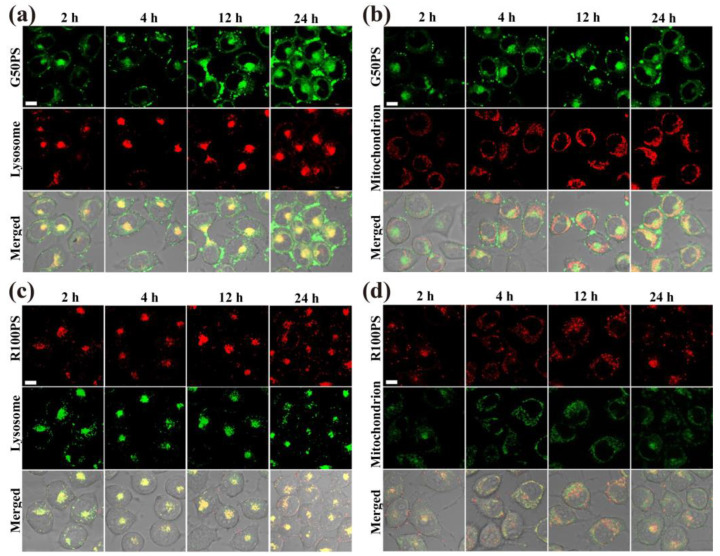
CLSM images of the subcellular location of PS NPs (20 μg/mL) in A549 cells after exposure to G50PS (green color) or R100PS (red color) for different times. Lysosomes and mitochondria are labeled red (**a**,**b**) or green (**c**,**d**). The scales represent 10 μm.

After entering cells via different pathways, the distribution of PS NPs in organelles varied depending on the size of PS NPs, cell type, and exposure conditions. As shown in Figure 5a,b, the fluorescence signal of G50PS in A549 increased with time. A major part of the fluorescence of G50PS overlapped with lysosomes (Figure 5a), and a small amount was distributed in mitochondria (Figure 5b). In addition, a certain number of G50PS were found around the cell membrane. One possible reason is that some G50PS entered A549 cells through caveolae-mediated endocytosis, which caused G50PS to be encapsulated in caveosomes and then delivered to endoplasmic reticulum other than lysosomes [46,56]. Additionally, some G50PS may enter A549 cells by penetrating the cell membrane through hydrophobic interaction and Van Der Waals’ force [53], as shown by weaker inhibition of the cellular uptake of G50PS at 4 °C (Figure 4a). At 24 h, some G50PS moved from lysosomes to mitochondria. Similarly, R100PS was mainly distributed in lysosomes and a small amount in mitochondria (Figure 5c,d); however, some R100PS left lysosomes in 24 h (Figure 5c) and probably moved to other organelles. A study on rat basophilic leukemia (RBL-2H3) cells reported that the internalized 50 nm PS NPs were mainly distributed in the lysosomes, and some signals were in the cytoplasm [53]. The phenomenon that PS NPs distributed in lysosomes can be delivered to other organelles, such as mitochondria, was also reported previously [57].

**Figure 6:**
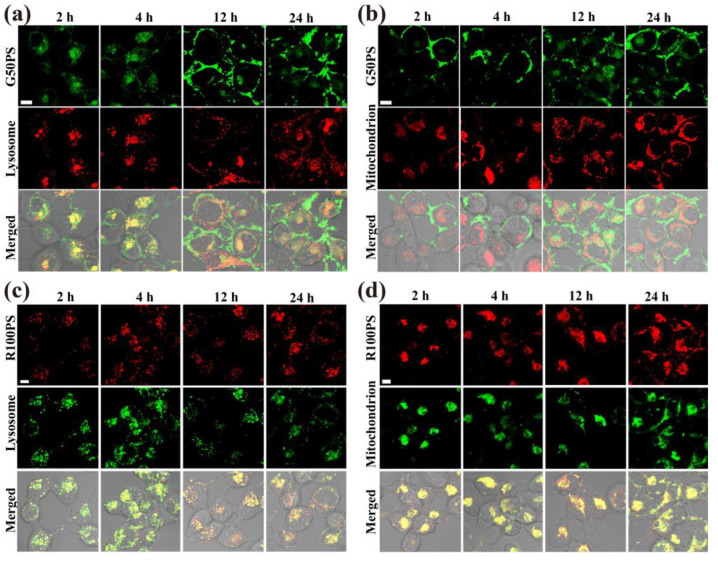
CLSM images of the subcellular location of PS NPs (20 μg/mL) in BEAS-2B cells after exposure to G50PS (green color) or R100PS (red color) for different times. Lysosomes and mitochondria are labeled red (**a**,**b**) or green (**c**,**d**). The scales represent 10 μm.

G50PS had a similar distribution profile in BEAS-2B cells as that in A549 cells, including distribution near the cell membrane and in lysosomes and mitochondria (Figure 6a,b), which were consistent with the endocytosis pathways of G50PS. In addition, some G50PS particles also left lysosomes, and a part of them began to transfer to mitochondria at 12 h. Interestingly, most R100PS were found in mitochondria and some in lysosomes in BEAS-2B cells within 4 h, and a part in mitochondria moved to lysosomes after 12 h (Figure 6c,d).

### 3.5. Exocytosis of PS NPs

**Figure 7:**
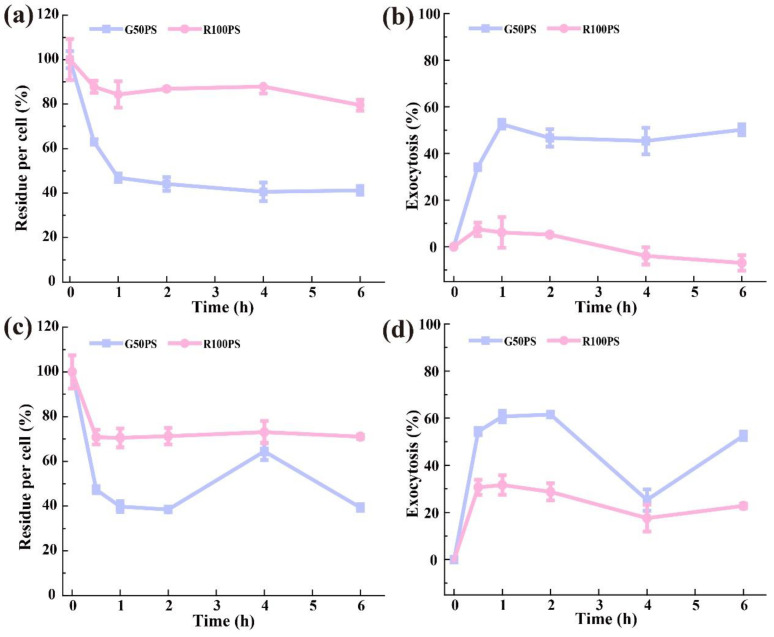
Exocytosis of PS NPs from cells after incubation with 20 μg/mL G50PS and R100PS for 4 h (n = 3). (**a**,**c**) Remaining contents of PS NPs in A549 cells (**a**) and BEAS-2B cells (**c**), as represented with the mean FI (% of control). (**b**,**d**) Exocytosis of PS NPs in A549 cells (**b**) and BEAS-2B cells (**d**).

After the cells had been exposed to the PS NPs for 4 h, the exocytosis of PS NPs was investigated in fresh medium. The results in Figure 7 show that the content of PS NPs in A549 cells decreased significantly in the first 1 h, followed by a slow decrease. One hour after excretion, the contents of G50PS and R100PS in A549 cells were close to 47% and 84% of the controls, respectively; the contents of G50PS and R100PS almost reached the minimum at 6 h, with values of 41% and 80%, respectively (Figure 7a). To eliminate the effect of cell division on the contents of PS NPs in cells, we counted the cells at different test points (Figure S9a) to calculate the total amount of PS NPs excreted outside cells. As shown in Figure 7b, the real external displacement of G50PS basically reached the maximum after 1 h, where about 53% of intracellular G50PS was discharged into culture medium. However, there was no significant exocytosis of R100PS within 6 h (Figure 7b), indicating R100 PS was difficult to be expelled from A549 cells. The higher content of G100PS at a longer time and the persistence of R100PS in cells suggest long-term damage to cells. 

The PS NPs in BEAS-2B cells showed a few different exocytosis characteristics. Both G50PS and R100PS also displayed the fastest discharge in the first 1 h and reached the maximum, where the contents of G50PS and R100PS were about 40% and 70% of the controls, respectively (Figure 7c). Surprisingly, the intracellular G50PS had an obvious change at 4 h, but there is no good explanation at present. After calibration with cell numbers (Figure S9b), it was found that about 53% of G50PS and 23% of R100PS were exocytosed from BEAS-2B cells within 6 h (Figure 7d). In both kinds of cells, the excretion was very fast within the first 1 h, followed by a slow process, and G50PS was easier to be excreted than R100PS, which is consistent with the results of other NPs [53,58].

### 3.6. Exocytosis Pathway of PS NPs

**Figure 8:**
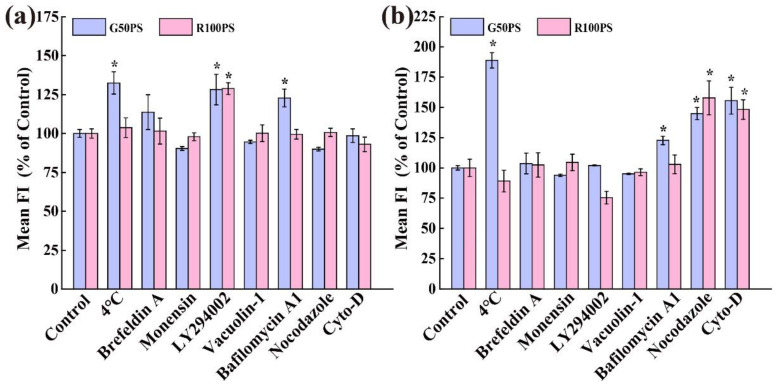
Exocytosis pathways of PS NPs in A549 cells (**a**) and BEAS-2B cells (**b**). Cells were incubated with 20 μg/mL PS NPs for 4 h, and then the cells were cultured in fresh medium at 4 °C or with/without exocytosis inhibitors for 2 h. The cells cultured in the fresh medium without exocytosis inhibitors were set as the control (100%). * *p* < 0.05 compared with the control (n = 3).

Again, we used low-temperature treatment and exocytosis inhibitors to reveal the exocytosis mechanisms of the PS NPs. As shown in Figure 8a, the exocytosis of G50PS was inhibited at 4 °C, indicating that the exocytosis of G50PS in A549 cells was energy-dependent. Additionally, Bafilomycin A1 and LY294002 significantly inhibited the discharge of G50PS from A549, inferring that lysosomes play an important role in the exocytosis process. For R100PS particles, only LY294002 significantly inhibited their efflux in A549 cells; that is, the lysosome pathway is also important for the exocytosis of R100PS, although only a small amount of R100PS could be exocytosed in A549 cells. However, both nocodazole and Cyto D had no inhibitory effect, suggesting that the exocytosis was independent of tubulin and actin. Clearly, the exocytosis of the PS NPs in A549 cells strongly depended on lysosomes but not tubulin and actin. In contrast, nocodazole and Cyto D had a vital inhibitory effect on the excretion of the PS NPs in BEAS-2B cells, suggesting that the efflux was highly dependent on tubulin and actin (Figure 8b). Moreover, the exocytosis of G50PS in BEAS-2B cells was almost completely inhibited at 4 °C (Figure 8b), indicating that the efflux was heavily energy-dependent. Bafilomycin A1 also slightly inhibited the efflux of G50PS, indicating lysosomes were involved in the exocytosis. It should be noted here that the exocytosis of R100PS in both kinds of cells was very small, and thus the exocytosis mechanism was difficult to investigate by using the inhibition methods.

**Figure 9:**
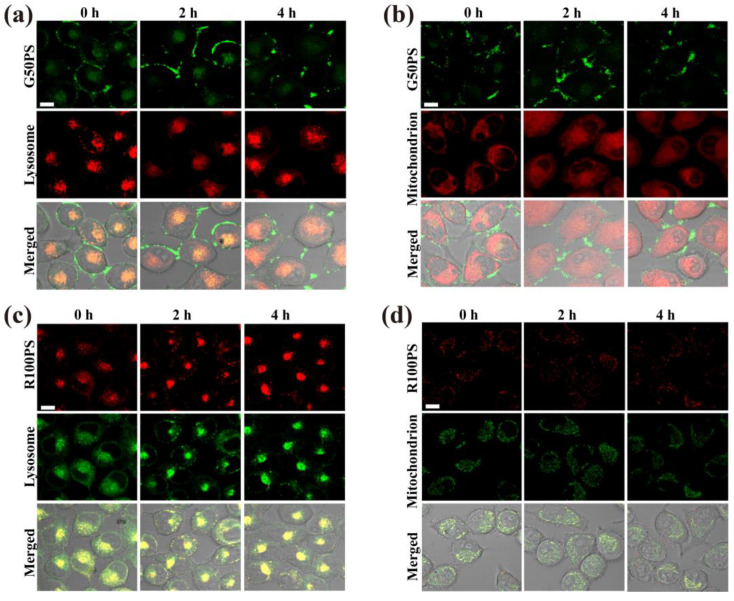
CLSM images of PS NPs in lysosomes (**a**,**c**) and mitochondrion (**b**,**d**) during exocytosis in A549 cells. Cells were pre-incubated with 20 μg/mL G50PS (**a**,**b**) and R100PS (**c**,**d**) for 4 h and then incubated in fresh culture medium for 0, 2, and 4 h. The scales represent 10 μm.

**Figure 10:**
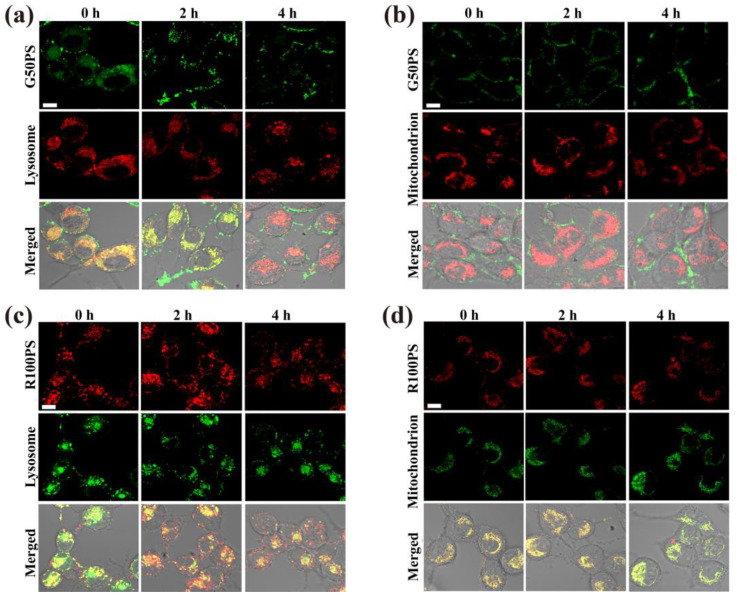
CLSM images of PS NPs in lysosomes (**a**,**c**) and mitochondrion (**b**,**d**) during exocytosis in BEAS-2B cells. Cells were pre-incubated with 20 μg/mL G50PS (**a**,**b**) and R100PS (**c**,**d**) for 4 h and then incubated in fresh culture medium for 0, 2, and 4 h. The scales represent 10 μm.

Furthermore, using CLSM, we observed that some G50PS in A549 cells left the lysosomes at 4 h (Figure 9a). However, no changes were observed in mitochondria (Figure 9b), possibly because of the low content of G50PS in mitochondria. The content of R100PS in both lysosomes and mitochondria did not change significantly (Figure 9c,d). Different from A549 cells, a significant decrease in G50PS in BEAS-2B cells over time was observed (Figure 10a), while there was no increased accumulation in mitochondria over time (Figure 10b), suggesting the exocytosis of G50PS via the lysosome pathway. As for R100PS, its contents in both lysosomes and mitochondria decreased, suggesting their translocation in cells and possible weak exocytosis (Figure 10c,d).

## 4. Conclusions

Our study demonstrated that PS NPs were internalized by human lung cells in a time-, dose-, particle size-, and cell type-dependent manner. The longer the incubation time and the higher the dose, the higher the cellular uptake of PS NPs. Moreover, A549 cells ingested more PS NPs than BEAS-2B cells did. Generally, the uptake of G50PS by human lung cells was easier than that of R100PS, but this observation was only valid under a certain time and concentration range. At higher concentrations and longer times, both kinds of lung cells could internalize more R100PS than G50PS based on mass. The internalization pathways of the PS NPs by the two kinds of cells varied dramatically. Lysosome was the main organelle for the two PS NPs in cells, while mitochondrion was an important organelle for R100PS in BEAS-2B cells, and some G50PS NPs were distributed around the cell membrane in both kinds of cells. It was found that the larger PS NPs were more difficult to excrete from the lung cells than the smaller ones. There was no obvious excretion of R100PS from A549 cells, while about 50% of the ingested G50PS was expelled within 6 h. The efflux of G50PS in A549 cells depended on lysosomes, and that in BEAS-2B cells depended on actin and microtubule. These findings deepen the acquaintance of the interaction of PS NPs with cells, which might help to evaluate the impact of nanoplastics pollution on human health.

## Figures and Tables

**Table 1:** Hydrodynamic sizes and ζ-potentials of G50PS and R100PS in aqueous solutions.

Dispersing Agent	Time (h)	Size (nm)		ζ-Potential (mV)	
		G50PS	R100PS	G50PS	R100PS
Water	0	40.3 ± 9.0	86.9 ± 20.5	–34.1 ± 10.8	–22.2 ± 11.5
	6	38.0 ± 9.4	88.7 ± 22.6	–38.7 ± 9.2	–18.7 ± 7.7
	12	41.2 ± 8.3	86.7 ± 24.0	–41.2 ± 10.8	–22.8 ± 11.4
Culture medium	0	62.8 ± 25.3	103.0 ± 26.4	–12.4 ± 4.1	–9.4 ± 5.3
	6	67.7 ± 25.6	113.9 ± 20.9	–10.8 ± 3.1	–8.4 ± 7.2
	12	64.6 ± 22.8	111.6 ± 19.8	–12.1 ± 2.8	–10.0 ± 3.5

## Data Availability

Data are available from the authors upon reasonable request.

## Supplementary Materials

The following supporting information can be downloaded at: https://www.mdpi.com/article/10.3390/nano13010084/s1, Figure S1: Fluorescence spectra of G50PS and R100PS; Figure S2: FIs of G50PS and R100PS at different concentrations in ultrapure water and culture medium; Figure S3: Fluorescence leakage from G50PS and R100PS incubated in culture medium at 37 °C for different time periods; Figure S4: Viability of A549 cells and BEAS-2B cells after exposure to PS NPs for 24 h; Figure S5: Cellular uptake of PS NPs after cells were incubated with different concentrations of PS NPs for 2 h; Figure S6: The external standard method was used to calibrate the cellular uptake of the PS NPs; Figure S7: Cellular uptake of PS NPs after cells were exposed to PS NPs (20 μg/mL) for different times; Figure S8: Viability of A549 cells and BEAS-2B cells after exposure to each endocytosis inhibitor for 4 h; Figure S9: Cell number of A549 cells and BEAS-2B cells during the exocytosis process of PS NPs; Figure S10: Viability of A549 cells and BEAS-2B cells after exposure to each exocytosis inhibitor for 4 h.
