# Supplementary material for: Endocytosis, Distribution, and Exocytosis of Polystyrene Nanoparticles in Human Lung Cells
Source: Nanomaterials (Basel). 2022 Dec 24;13(1):84. doi: 10.3390/nano13010084 (PMC9824409; doi:10.3390/nano13010084)
Supplement: Supplementary file 1 [file nanomaterials-13-00084-s001.zip › nanomaterials-2115331-supplementary.pdf]

nanomaterials

Supplementary Materials

Endocytosis, Distribution, and Exocytosis of Polystyrene Nanoparticles in Human Lung Cells

Yuan-Yuan Liu[1#], Jie Liu[1#], Hao Wu[1], Qiangqiang Zhang[1], Xue-Rui Tang[1], Dan Li[1], Chen-Si Li[1], Yuanfang Liu[1,2], Aoneng Cao[1,*] and Haifang Wang[1,*]

[1] Institute of Nanochemistry and Nanobiology, Shanghai University, Shanghai 200444, China
[2] Beijing National Laboratory for Molecular Sciences, College of Chemistry and Molecular Engineering, Peking University, Beijing 100871, China
# These authors contribute equally.
* Correspondence: hwang@shu.edu.cn (H. Wang), ancao@shu.edu.cn (A. Cao)

This PDF file includes: Figures S1–S10.

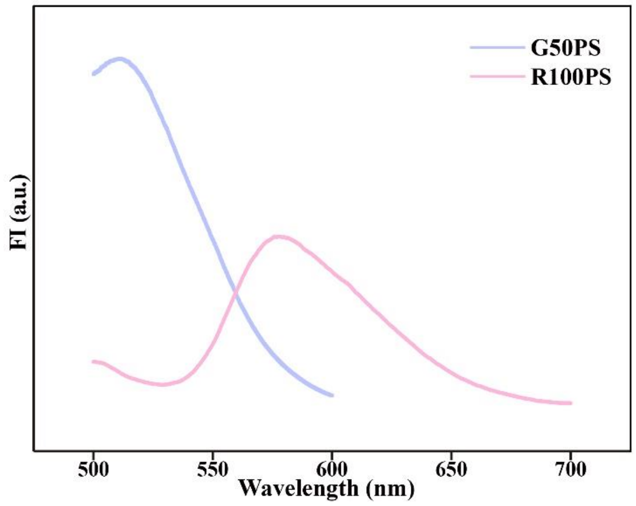

Figure S1. Fluorescence spectra of G50PS and R100PS (Ex 488 nm).

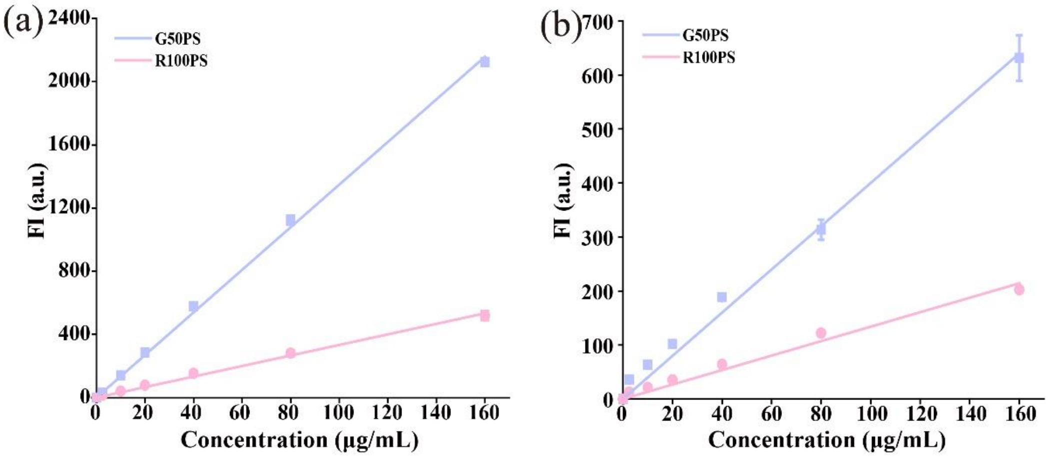

Figure S2. FIs of G50PS (Ex 488 nm/Em 508 nm) and R100PS (Ex 488 nm/Em 578 nm) at different concentrations in ultrapure water **(a)** and culture medium **(b)** (n=3).

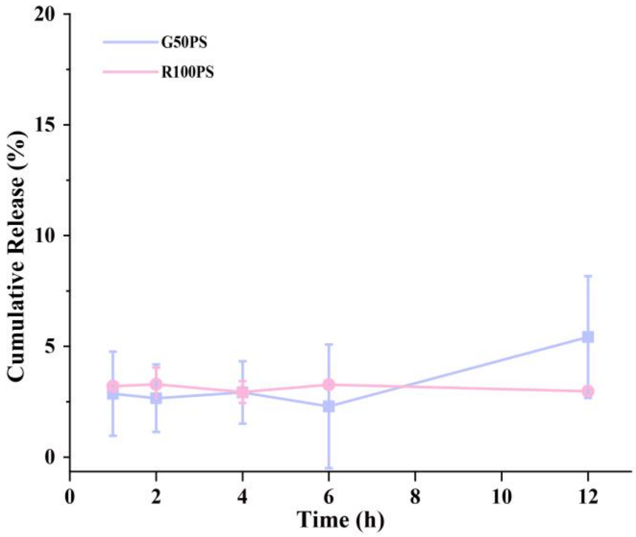

Figure S3. Fluorescence leakage from G50PS and R100PS incubated in culture medium at 37 °C for different time periods (n=3).

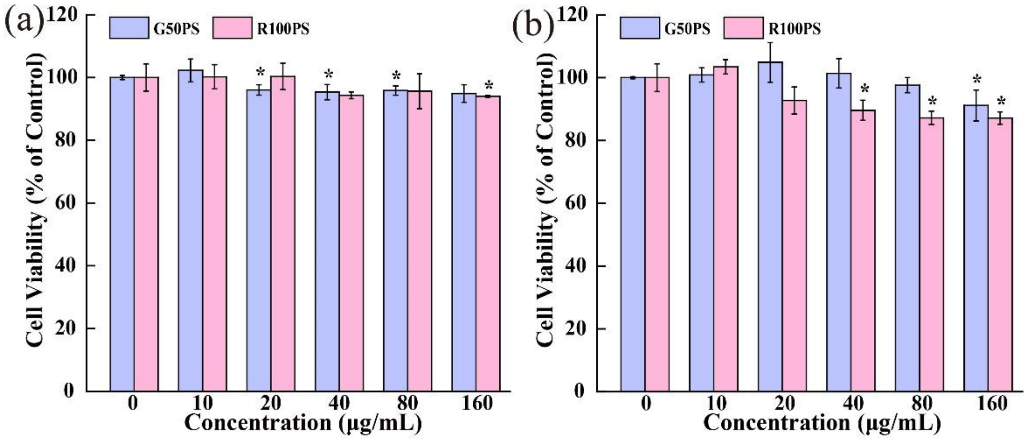

Figure S4. Viability of A549 cells **(a)** and BEAS-2B cells **(b)** after exposure to PS NPs for 24 h. *P < 0.05 compared with the control (n=3).

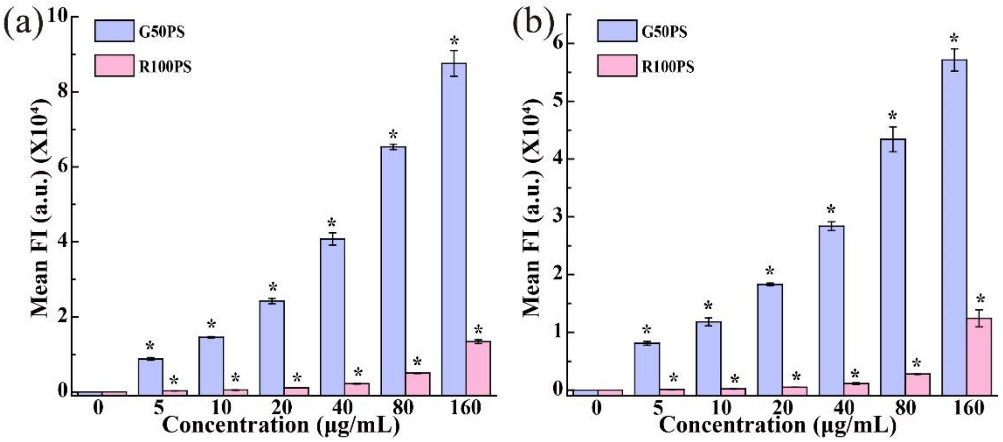

Figure S5. Cellular uptake of PS NPs after cells were incubated with different concentrations of PS NPs for 2 h. The mean FIs of A549 cells **(a)** and BEAS-2B cells **(b)** were measured by flow cytometry. *P < 0.05 vs the control (n = 3).

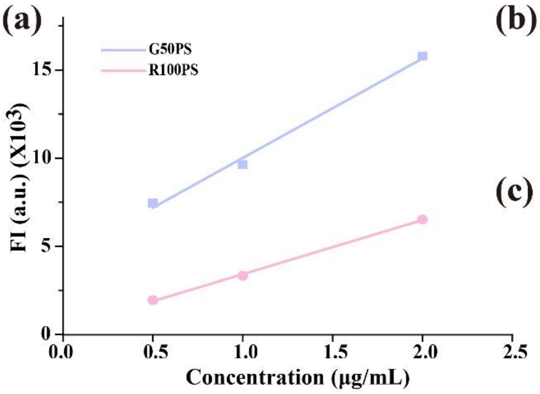

(b)

FS5	Uptake (μg/mL)
G50PS	0.42
R100PS	0.14

(c)

Flow cytometry	FI		Ratio (G50PS/R100PS)
	Mean	Correction	
G50PS	62128.67	147925.41	11.85
R100PS	1747.63	12483.07	

Figure S6. The external standard method was used to calibrate the cellular uptake of the PS NPs by flow cytometry. **(a)** FIs of G50PS (Ex 488 nm/Em 508 nm) and R100PS (Ex 488 nm/Em 578 nm) of different concentrations in the cell lysate samples. BEAS-2B cells were exposed to 20 μg/mL PS NPs for 4 h. The FIs were measured by the spectrometer FS5. **(b)** Cellular uptake of the PS NPs calculated based on the data in (a). **(c)** Calibration ratio of the fluorescence results of G50PS (Ex 488 nm/Em 508 nm) and R100PS (Ex 488 nm/Em 578 nm) measured by flow cytometry.

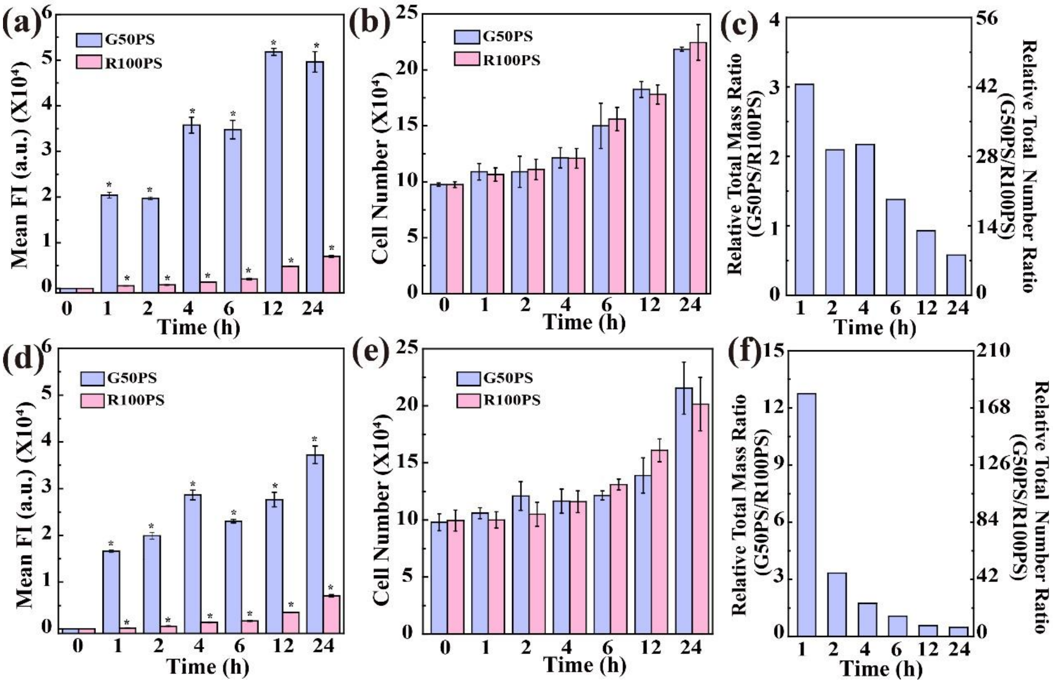

Figure S7. Cellular uptake of PS NPs after cells were exposed to PS NPs (20 μg/mL) for different times. (**a** and **d**) Mean FIs in A549 cells **(a)** and BEAS-2B cells **(d)**. (**b** and **e**) Cell numbers of A549 cells **(b)** and BEAS-2B cells **(e)**. (**c** and **f**) Relative total mass (left y-axis)/number (right y-axis) uptake ratios (G50PS/R100PS) of A549 cells **(c)** and BEAS-2B cells **(f)**. *$P < 0.05$ compared with the control (n=3).

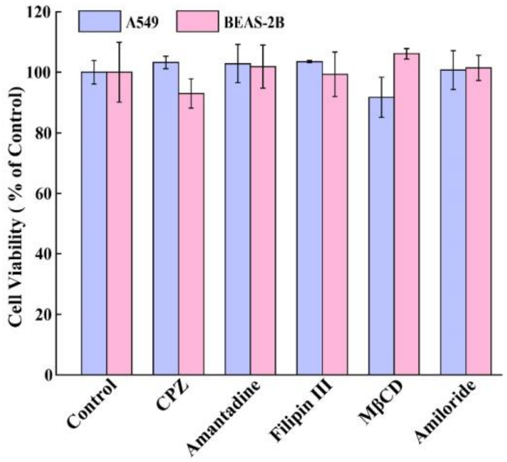

Figure S8. Viability of A549 cells and BEAS-2B cells after exposure to each endocytosis inhibitor for 4 h (n=3).

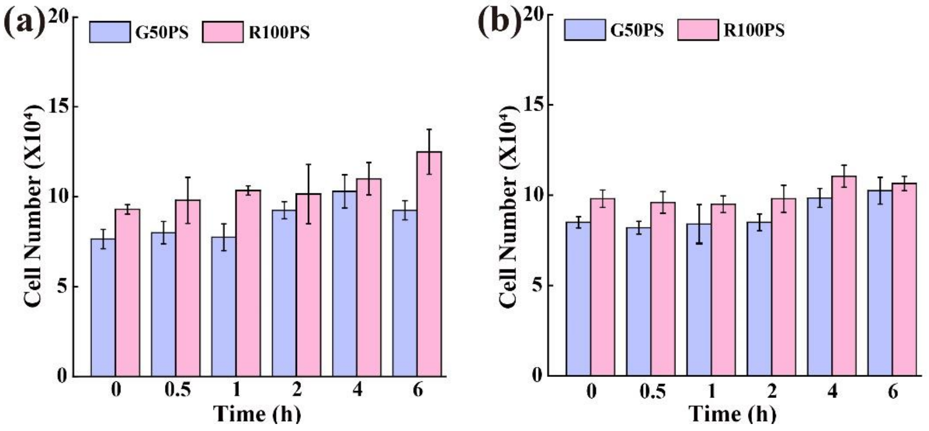

Figure S9. Cell number of A549 cells **(a)** and BEAS-2B cells **(b)** during the exocytosis process of PS NPs. (n=3).

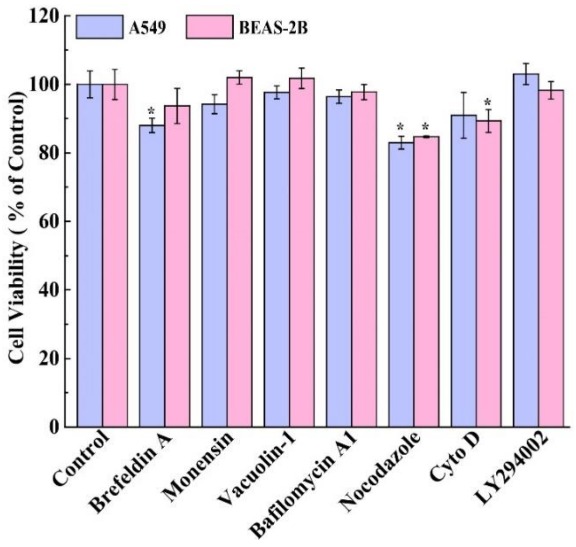

Figure S10. Viability of A549 cells and BEAS-2B cells after exposure to each exocytosis inhibitor for 4 h (n=3).
